# Water Extract of *Dryopteris crassirhizoma* Attenuates Bone Loss by Suppressing Osteoclast Differentiation and Function

**DOI:** 10.1155/2013/852648

**Published:** 2013-05-20

**Authors:** Hyunil Ha, Ki-Shuk Shim, Taesoo Kim, Hyosun An, Jin Yeul Ma

**Affiliations:** KM-Based Herbal Drug Research Group, Korea Institute of Oriental Medicine, Daejeon 305-811, Republic of Korea

## Abstract

The rhizome of *Dryopteris crassirhizoma* has been used as a traditional herbal medicine for treating various inflammatory and infectious diseases such as tapeworm infestation and mumps. In the present study, we investigated the bone protective effect of water extract of the rhizome of *Dryopteris crassirhizoma* (WEDC). We found that WEDC inhibits osteoclast differentiation via directly acting on osteoclast precursors. In osteoclast precursors, WEDC inhibited receptor activator of nuclear factor-**κ**B ligand- (RANKL-) induced expression of c-Fos and nuclear factor of activated T cells cytoplasmic 1, a key downstream target of c-Fos during osteoclast differentiation. We found that WEDC inhibits RNAKL-induced activation of extracellular-regulated kinase and NF-**κ**B that mediates c-Fos expression and osteoclast differentiation. In addition to the inhibitory effect of osteoclast differentiation, WEDC markedly suppressed bon-resorbing activity of mature osteoclasts, which was accompanied by disruption of actin ring structure. Furthermore, administration of WEDC suppressed RANKL-induced trabecular bone loss in mice. Collectively, our results demonstrate that WEDC inhibits not only osteoclast differentiation by inhibiting RANK signaling pathways in osteoclast precursors but also bone resorption by disrupting actin ring in mature osteoclasts, thereby contributing to its protective effect on bone loss.

## 1. Introduction

Bone homeostasis is maintained by the coordinated actions of osteoclast-mediated bone resorption and osteoblast-mediated bone formation. An imbalance in favor of bone resorption, most often due to excess osteoclastic activity, leads to bone loss in pathological conditions such as osteoporosis, lytic bone metastases, and rheumatoid arthritis [1]. 

Osteoclasts are multinucleated bone-resorbing cells that originate from hematopoietic mononuclear precursors. Receptor activator of nuclear factor-*κ*B ligand (RANKL) is an essential cytokine that stimulates entire processes for the development of bone-resorbing osteoclasts [1]. The effects of RANKL are physiologically blocked by osteoprotegerin (OPG) which acts as a decoy receptor for RANKL [2]. Stromal cells/osteoblasts regulate osteoclast differentiation by expressing both RANKL and OPG [1]. Thus, RANKL/OPG ratio in osteoblasts is an important determinant of osteoclast differentiation as well as bone mass. RANKL binding to its receptor RANK on osteoclast precursors induces activation of NF-*κ*B and mitogen-activated protein kinases (MAPKs) including extracellular-regulated kinase (ERK), c-Jun N-terminal kinase (JNK), and p38 through recruitment of the adapter molecules such as TNF receptor-associated factor 6 (TRAF6) [3, 4]. These signaling cascades lead to the induction and activation of osteoclastogenic transcription factors such as c-Fos and nuclear factor of activated T cells cytoplasmic 1 (NFATc1) [4–8]. Genetic studies have shown that deficiency in NF-*κ*B p50/p52, c-Fos, or NFATc1 results in blockage of osteoclastogenesis [9–11]. 

In the screening of traditional Korean herbal medicines for antiosteoclastogenic activity, we found that water extract of the rhizome of *Dryopteris crassirhizoma* (WEDC) efficiently inhibits osteoclast differentiation. *D. crassirhizoma *is a perennial herbaceous plant widely distributed in Asia, and its rhizome is commonly used as an effective vermicide in traditional Chinese medicine. It has also been prescribed for treating respiratory tract infections, uterine bleeding, mumps, and feverish illnesses [12]. Previous studies have shown that the rhizome of *D. crassirhizoma *has antioxidant, anticancer, anti-inflammatory, and antibacterial activities [13–16]. However, there is little information about the pharmacological effect of the rhizome of *D. crassirhizoma* on bone metabolism. In the present study, we investigated the inhibitory effect and action mechanism of WEDC on osteoclast differentiation and function. The *in vivo* efficacy of WEDC was also examined in a murine model of bone loss.

## 2. Materials and Methods

### 2.1. Reagents and Antibodies

*α*-MEM and FBS were purchased from Thermo Fisher Scientific Inc. (Rockford, IL, USA). Phalloidin-TRITC, 1*α*,25-dihydroxyvitamin D3 (VitD_3_), and *p*-nitrophenyl phosphate were purchased from Sigma-Aldrich (St. Louis, MO, USA). Recombinant human M-CSF was kindly provided by Dr. Yongwon Choi (University of Pennsylvania, School of Medicine). Recombinant human soluble RANKL was prepared as described in a previous report [17]. Antibodies against phospho-ERK1/2 (thr202/Tyr204), ERK, phospho-JNK1/2 (Thr183/Tyr185), JNK, phospho-p38 (Thr180/Tyr182), p38, phospho-p65 (Ser536), p65, phospho-I*κ*B*α* (Ser32), and I*κ*B*α* were purchased from Cell Singling Technology (Danvers, MA, USA). Antibodies against NFATc1, c-Fos, *β*-actin, and GAPDH were from Santa Cruz Biotechnology (Santa Cruz, CA, USA). 

### 2.2. Preparation of WEDC

The rhizome of *D. crassirhizoma* was purchased from Yeongcheon Oriental Herbal Market (Yeongcheon, Republic of Korea). A voucher specimen (no. W197) was deposited in the herbal bank of KM-Based Herbal Drug Research Group, Korea Institute of Oriental Medicine. The rhizome of *D. crassirhizoma* (50 g) was extracted with 500 mL of boiling water for 3 h. The extract was filtered through a testing sieve (150 *μ*m) and lyophilized in a freeze dryer. To prepare WEDC, the lyophilized powder (yield: 12.78%) was resuspended in distilled water, centrifuged at 10,000 ×g for 5 min, and filtered through a 0.2 *μ*m sterile filter. 

### 2.3. Cell Culture and Osteoclast Differentiation

Bone-marrow-derived macrophages (BMMs) were prepared from mouse bone marrow cells as described previously [18] and cultured in *α*-MEM complete medium containing 10% FBS, antibiotics (100 U/mL penicillin and 100 *μ*g/mL streptomycin), and M-CSF (60 ng/mL). To generate osteoclasts from BMM cultures, BMMs (1 × 10^4^ cells/well) were incubated with M-CSF (60 ng/mL) and RANKL (100 ng/mL) for 4 days in 96-well plates. Cell viability of BMMs was measured with Cell Counting Kit-8 assay (Dojindo Molecular Technologies Inc., Rockville, MD, USA), after culturing BMMs (1 × 10^4^ cells/well in a 96-well plate) in the presence of M-CSF (60 ng/mL) for 2 days. Mouse calvarial osteoblasts were obtained from calvariae of newborn mice as described previously [18]. For osteoclast differentiation from the coculture of mouse osteoblasts and bone marrow cells, bone marrow cells (3 × 10^5^ cells/well) and osteoblasts (2 × 10^4^ cells/well) were cocultured with 10 nM VitD_3_ for 6 days in 48-well tissue culture plates. All cultures were replenished with fresh medium on day 3.

### 2.4. Tartrate Resistant Acid Phosphatase (TRAP) Activity and Staining

Cells were fixed in 10% neutral buffered formalin and permeabilized with 0.1% Triton X-100. Total TRAP activity was measured spectrophotometrically using *p*-nitrophenyl phosphate as substrate according to the manufacturer's instructions in a TRAP assay buffer (50 mM sodium tartrate and 0.12 M sodium acetate, pH 5.2). TRAP staining was performed according to the protocol described in BD Biosciences Technical Bulletin no. 445. TRAP-positive multinucleated cells (TRAP(+)MNCs) having more than three nuclei were counted as osteoclasts.

### 2.5. Real-Time Quantitative Polymerase Chain Reaction (QPCR)

Osteoblasts (3 × 10^5^ cells/well in a 6-well plate) were preincubated with WEDC for 3 h and then stimulated with VitD_3_ for 24 h. BMMs (4 × 10^5^ cells/well in a 6-well plate) were incubated with WEDC for 3 h and then stimulated with RANKL for the indicated times. Total RNA was isolated using RNA-spin total RNA Extraction Kit (Bioneer, Daejeon, Republic of Korea), and cDNA was synthesized from 1 *μ*g of total RNA using AccuPower RT-PreMix (Bioneer). SYBR green-based QPCR amplification was performed using cDNA diluted to 1 : 3, 10 pmol of primers, and AccuPower GreenStar QPCR Master Mix (Bioneer) in the Applied Biosystems 7500 Real-Time PCR System (Applied Biosystems, Foster City, CA, USA). The primer sequences used were described previously [18]. The relative mRNA levels of target genes were determined according to the 2-ΔΔCT method using Hprt as a reference gene. 

### 2.6. Western Blot Analysis

Cells were washed twice with ice-cold PBS and lysed in ice-cold lysis buffer (20 mM Tris-HCl, 150 mM NaCl, 1 mM EDTA, 1 mM EGTA, 1% NP-40, and 0.1% SDS) supplemented with protease inhibitor and phosphatase inhibitor cocktail tablets (Roche Applied Science, Indianapolis, IN, USA). Cell lysates were centrifuged at 10,000 ×g for 15 min at 4°C. Protein concentration of total cell lysates was determined by suing BCA protein assay kit (Thermo Fisher Scientific Inc.). Equal amounts of proteins (30 *μ*g) were subjected to sodium dodecyl sulfate-polyacrylamide gel electrophoresis, transferred to a polyvinylidene fluoride membrane, and then immunoblotted with the indicated antibodies.

### 2.7. Bone Resorption Assay

Mature osteoclasts were generated by coculturing mouse bone marrow cells and primary osteoblasts with VitD_3_ (10 nM) on collagen gels for 6 days. The generated osteoclasts were seeded on an Osteo Assay Surface plate (Corning Inc., Corning, NY, USA), allowed to settle for 2 h, and then incubated with the indicated concentrations of WEDC for 24 h. Cells were stained for TRAP to detect osteoclasts and photographed. Resorbed pits by osteoclasts were photographed and analyzed by using Image J software, after removing cells with sodium hypochlorite bleach.

### 2.8. Actin Ring Assay

BMMs were incubated with M-CSF and RANKL for 4 days to generate osteoclasts as described above. The cultures were treated with vehicle or the indicated concentrations of WEDC for 30 min, fixed, permeabilized, and then incubated with phalloidin-TRITC (0.2 *μ*g/mL in PBS) to stain F-actin. The peripheral actin ring-like structures in osteoclasts were photographed using a fluorescence microscope (Olympus IX53 inverted microscope).

### 2.9. *In Vivo* Experiments

Eight-week-old male ICR mice (6 mice/group) were orally administrated with distilled water or WEDC (0.25 g/kg of body weight twice daily) for 5 days. RANKL (1 mg/kg of body weight) or PBS was intraperitoneally injected on days 3 and 4. The mice were sacrificed on day 6, and the right femurs were dissected, cleaned of soft tissue, and fixed in 10% neutral buffered formalin. Microcomputed tomography (micro-CT) was performed with the SMX-90CT system (90 kVp, 109 mA, and 180-ms integration time; Shimadzu, Kyoto, Japan). Scans then were integrated into 3D voxel images. All bone images were reconstructed by the VG Studio MAX 1.2.1 program (Volume Graphics, Heidelberg, Germany). The regenerated trabecular bone volume/tissue volume, number, thickness, and separation were calculated with TRI/3D-BON (RATOC System Engineering, Kyoto, Japan). All animal experiments were performed according to the Guide for the Care and Use of Laboratory Animals of the National Institutes of Health. The experimental protocols were approved by the Institutional Animal Care and Use Committee at Korea Institute of Oriental Medicine.

### 2.10. Statistical Analysis

All values are presented as mean ± SD (*n* ≥ 3). Two-group comparisons were performed with Student's *t*-tests, while multiple-group comparisons were performed with analysis of variance followed by Dunnett's test. A *P* value <0.05 was considered statistically significant.

## 3. Results

### 3.1. Effect of WEDC on Osteoclast Differentiation in Bone Marrow Cell-Osteoblast Coculture

**Figure 1:**
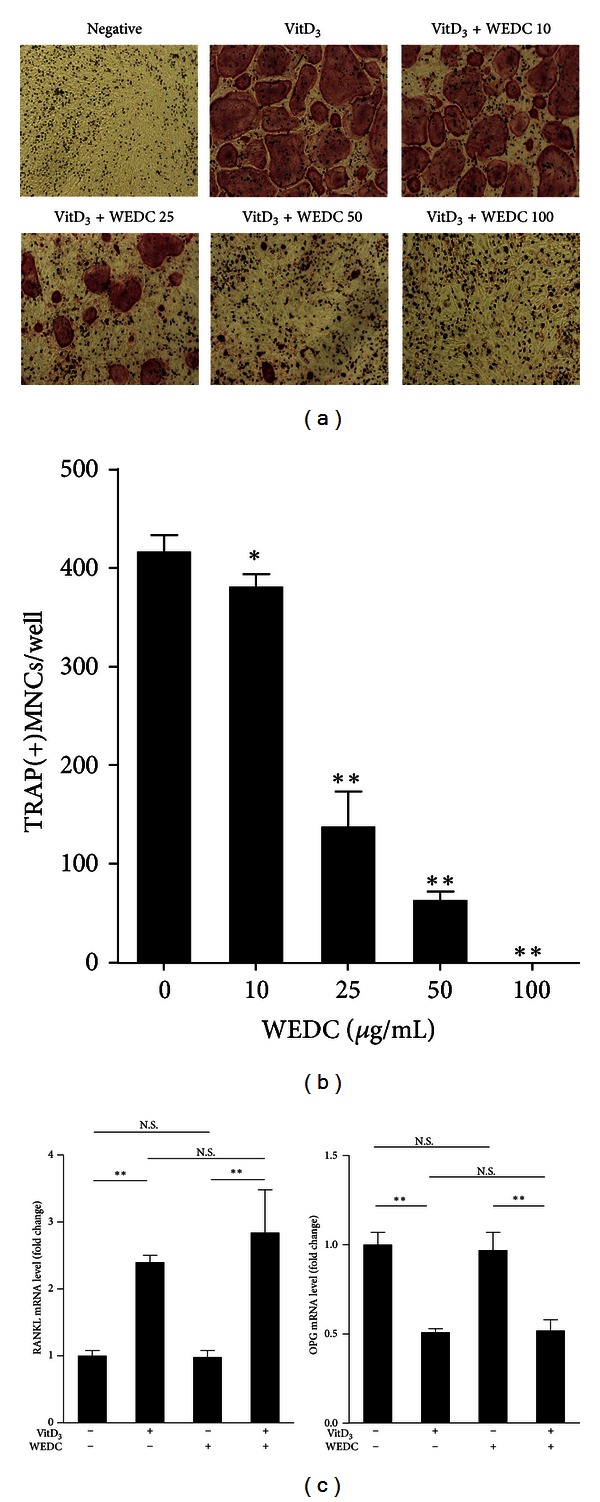
WEDC inhibits osteoclast differentiation in bone marrow cell-osteoblast coculture. Mouse bone marrow cells and calvarial osteoblasts were cocultured in the presence or absence (negative) of VitD_3_ (10 nM) with vehicle (distilled water) or WEDC (10, 25, 50, and 100 *μ*g/mL) for 6 days. (a) Representative microscopic pictures of TRAP staining are shown. (b) The number of TRAP(+)MNCs containing three or more nuclei were counted. Data are expressed as mean ± SD and are representative of three experiments. *P < 0.05 and **P < 0.01 versus vehicle-treated control group. (c) Osteoblasts were preincubated with vehicle or WEDC (50 *μ*g/mL) for 3 h and stimulated with VitD_3_ (10 nM) for 24 h. RANKL and OPG mRNA levels were analyzed by QPCR. Data are expressed as mean ± SD and are representative of three experiments. **P < 0.01. N.S.: not significant.

We investigated whether WEDC affects osteoclast differentiation in bone marrow cell-osteoblast coculture system. In the coculture system, osteoclastogenic factors such as VitD_3_ and IL-1 stimulate osteoclast differentiation by increasing the RANKL/OPG expression ratio in osteoblasts [1]. VitD_3_ stimulated osteoclast differentiation in the coculture, which was inhibited by WEDC in a dose-dependent manner (Figures 1(a) and 1(b)). However, WEDC (50 *μ*g/mL), a concentration that markedly inhibited osteoclast differentiation in the coculture system, did not affect RANKL and OPG mRNA expression in basal and VitD_3_-stimulated osteoblasts (Figure 1(c)).

### 3.2. Effect of WEDC on RANKL-Induced Osteoclast Differentiation

**Figure 2:**
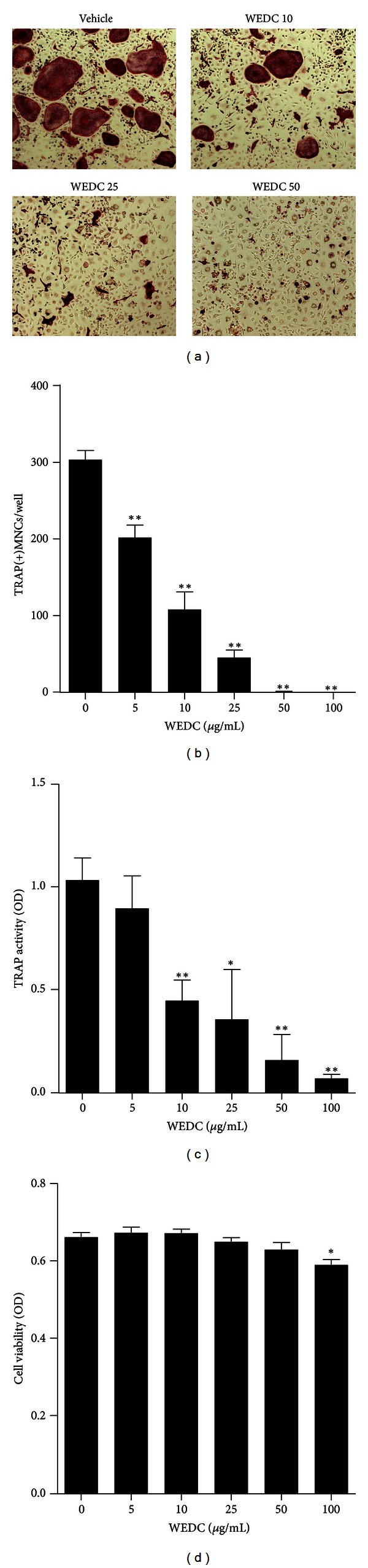
WEDC inhibits RANKL-induced osteoclast differentiation in BMMs. BMMs were incubated in the presence of M-CSF (60 ng/mL) and RANKL (100 ng/mL) with vehicle or WEDC (5, 10, 25, 50, and 100 *μ*g/mL) for 4 days. (a) Representative microscopic pictures of TRAP staining. (b) The number of TRAP(+)MNCs having more than three nuclei and (c) total cellular TRAP activity. (d) Effect of WEDC on the viability of BMMs was evaluated by Cell Counting Kit-8 assay. Data are expressed as mean ± SD and are representative of three experiments. *P < 0.05 and **P < 0.01 versus vehicle-treated control group.

We next investigated the possibility that WEDC inhibits osteoclast differentiation by directly acting on osteoclast precursors. In the presence of M-CSF, RANKL stimulated TRAP activity and osteoclast differentiation in its precursor BMM cultures. Consistent with the results of coculture system, WEDC inhibited RANKL-induced osteoclast differentiation and TRAP activity in a dose-dependent manner with an almost complete inhibition of osteoclast formation at 50 *μ*g/mL of WEDC (Figures 2(a)–2(c)). Although a slight reduction of cell viability of BMMs was observed at 100 *μ*g/mL of WEDC, 5–50 *μ*g/mL of WEDC did not affect cell viability of BMMs, indicating that the inhibitory effect of WEDC was not due to cellular toxicity or cell proliferation (Figure 2(d)).

### 3.3. Effect of WEDC on RANKL-Induced c-Fos and NFATc1 Expression in Osteoclast Precursors

**Figure 3:**
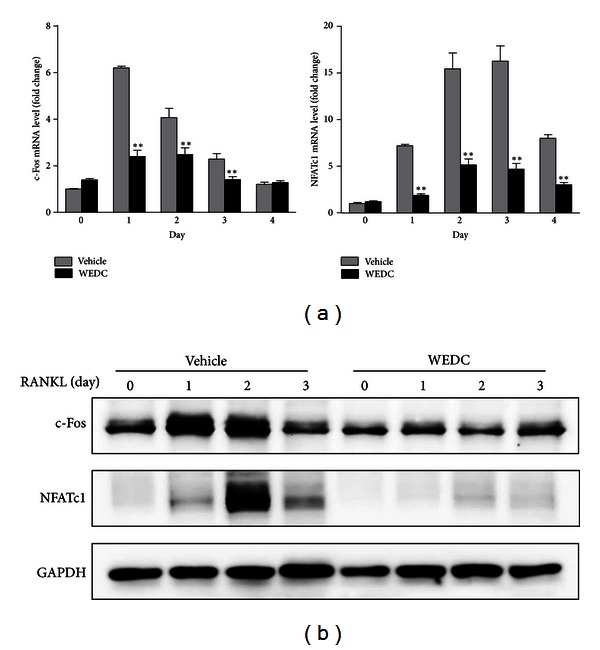
WEDC abrogates RANKL-induced c-Fos and NFATc1 expression in BMMs. BMMs were pretreated with or without WEDC (50 *μ*g/mL) for 3 h and cultured in the presence of M-CSF (60 ng/mL) and RANKL (100 ng/mL) for 4 days. Total RNA and cell lysates were isolated at the indicated time points. (a) mRNA levels of c-Fos and NFATc1 were analyzed by QPCR. Data are expressed as mean ± SD and are representative of three experiments. **P < 0.01 versus vehicle-treated control group. (b) Total cell lysates were analyzed by Western blot analysis with the indicated antibodies. GAPDH was used as a loading control.

NFATc1, the master transcription factor for osteoclastogenesis, is induced during osteoclastogenesis at a transcription level, and its induction is dependent on c-Fos [11]. To gain insights into the molecular mechanisms for the antiosteoclastogenic effect of WEDC, we determined whether WEDC affects c-Fos and NFATc1 expression during osteoclast differentiation. Stimulation of BMMs with RANKL increased c-Fos and NFATc1 expression at both mRNA and protein levels, which was dramatically suppressed by WEDC (Figures 3(a) and 3(b)).

### 3.4. Effect of WEDC on RANKL-Induced Early Signaling Pathways

**Figure 4:**
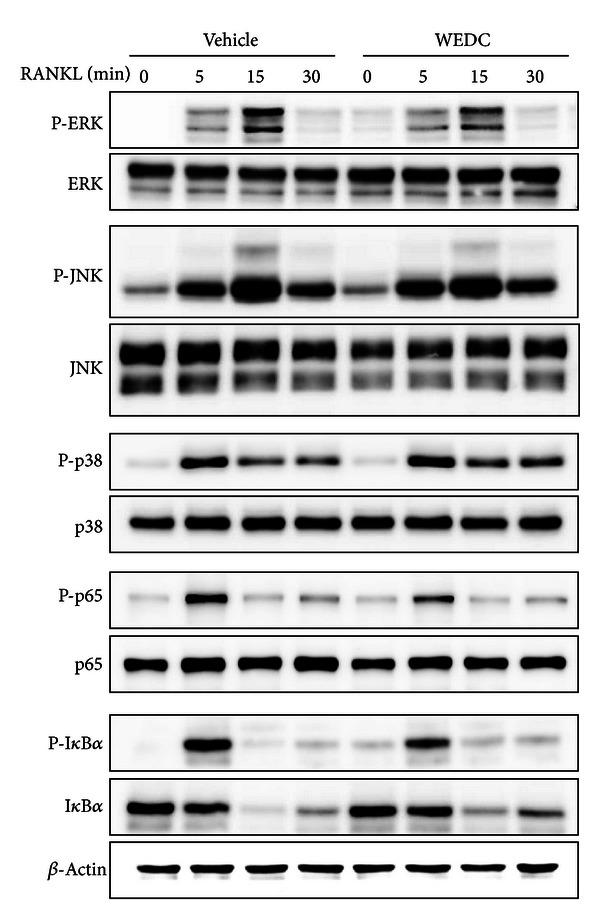
WEDC suppresses RANKL-induced activation of ERK and NF-*κ*B in BMMs. BMMs were pretreated with or without WEDC (50 *μ*g/mL) for 3 h and then stimulated with RANKL (100 ng/mL). Total cell lysates were prepared at the indicated time points and then analyzed by Western blot analysis with the indicated antibodies. *β*-Actin was used as a loading control.

MAPK and NF-*κ*B signaling pathways are implicated in RANKL-induced c-Fos and NFATc1 expression [5–8]. To elucidate molecular mechanisms underlying the inhibitory effect of WEDC on c-Fos and NFATc1 expression, we explored whether WEDC affects MAPK and NF-*κ*B activation in response to RANKL. Stimulation of BMMs with RANKL rapidly activated ERK, JNK, and p38 MAPKs as well as NF-*κ*B signaling pathway (p65 phosphorylation and I*κ*B*α* phosphorylation and degradation). WEDC slightly inhibited RANKL-induced ERK activation but not JNK or p38 activation. It also attenuated RANKL-induced p65 phosphorylation and I*κ*B*α* phosphorylation and degradation (Figure 4). 

### 3.5. Effect of WEDC on Bone-Resorbing Activity of Mature Osteoclasts

**Figure 5:**
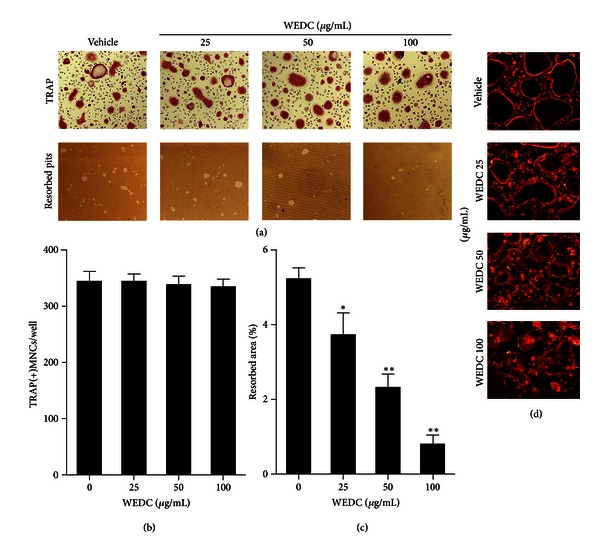
WEDC decreases bone-resorbing activity of mature osteoclasts. Mature osteoclasts were cultured with vehicle or WEDC on an Osteo Assay Surface plate for 24 h. (a) Representative microscopic pictures of TRAP staining (upper panel) and resorbed pits (down panel). (b) The number of TRAP(+)MNCs having more than three nuclei. (c) The total areas of resorbed pits. Data are expressed as mean ± SD and are representative of three experiments. *P < 0.05 and **P < 0.01 versus vehicle-treated control. (d) Mature osteoclasts were treated with vehicle or WEDC for 30 min, and F-actin was visualized with fluorescence microscopy.

We next investigated the effect of WEDC on osteoclastic bone resorption. When mature osteoclasts were cultured for 24 h on a plate coated with an inorganic crystalline calcium phosphate, numerous resorbed pits by osteoclasts were formed in vehicle-treated control cultures. WEDC decreased the total resorbed area in a dose-dependent manner, without affecting the number of osteoclasts (Figures 5(a)–5(c)). 

Actin ring is a distinctive cytoskeletal structure in mature osteoclasts, and its formation is essential for osteoclastic bone resorption [19]. Osteoclasts formed a ring-like F-actin structure, actin ring, at the cell periphery on tissue culture plates. Treatment of osteoclasts with WEDC for 30 min resulted in disruption of actin ring structure in a dose-dependent manner (Figure 5(d)).

### 3.6. Effect of WEDC on RANKL-Induced Bone Loss

**Figure 6:**
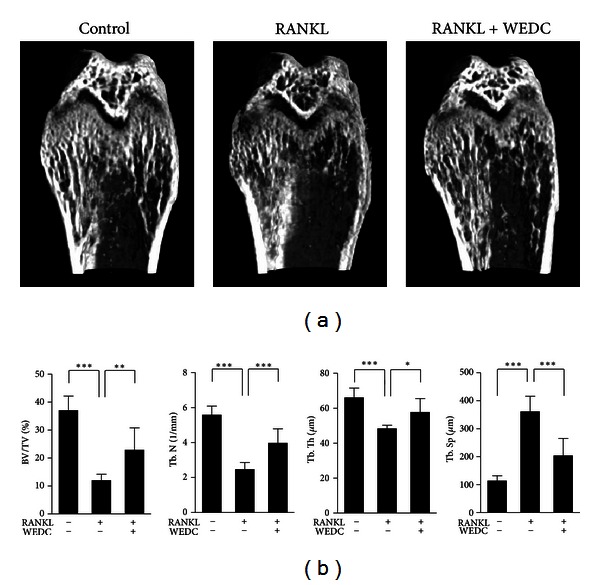
WEDC attenuates RANKL-induced bone loss in mice. 8-week-old mice were orally administrated with WEDC (0.25 g/kg) twice a day for 5 days, and RANKL (1 mg/kg) were intraperitoneally injected on days 3 and 4. The femora were collected on day 6 and analyzed by micro-CT scanning. (a) Representative micro-CT images of the distal femora. (b) Trabecular bone volume/tissue volume (BV/TV), trabecular number (Tb. N), trabecular thickness (Tb. Th), and trabecular separation (Tb. Sp) at the distal femoral metaphysis. Data are expressed as mean ± SD. *P < 0.05, **P < 0.01, ***P < 0.001.

Since WEDC exhibited the inhibitory activity on RANKL-induced osteoclast differentiation as well as bone-resorbing activity of mature osteoclasts, we next investigated whether WEDC has a protective effect on bone loss. When mice were intraperitoneally injected with RANKL twice with a 24 h interval, a severe trabecular bone loss was observed at the distal femoral metaphysis (Figure 6(a)). Micro-CT analysis revealed marked reduction in trabecular volume, thickness, and number, with a decreased trabecular separation in the RANKL-injected mice (Figure 6(b)). Oral administration of WEDC significantly attenuated RANKL-induced bone loss and the changes of bone structural parameters (Figures 6(a) and 6(b)).

## 4. Discussion

Abnormal activation of osteoclasts attributes to bone loss in many bone destructive diseases including postmenopausal osteoporosis, lytic bone metastases, and rheumatoid arthritis [1]. Accordingly, modulation of osteoclast differentiation and function can be a potent therapeutic target for various bone diseases characterized by excessive bone resorption. Here we have demonstrated for the first time that WEDC attenuates bone loss by suppressing osteoclast differentiation and function.

To explore the molecular mechanism by which WEDC inhibits osteoclast differentiation, we examined the RANKL/RANK/OPG system. WEDC did not affect RANKL and OPG expression in osteoblasts but inhibited the differentiation of osteoclast precursors into mature osteoclasts induced by RANKL stimulation. We found that WEDC inhibits RANKL-induced expression of c-Fos and NFATc1, a key downstream target of c-Fos during osteoclast differentiation, in osteoclast precursors.

Previous studies have shown that activation of MAPKs (ERK, JNK, and p38) and NF-*κ*B mediates RANKL-induced c-Fos expression and osteoclast differentiation [5–8]. It was reported that RANKL activates JNK, p38, and the classical NF-*κ*B signaling pathway through the RANK-TRAF6 pathway in a manner dependent on E3 ubiquitin ligase activity of TRAF6 [3, 4]. However, it is still unclear how RANKL activates ERK. In the present study, WEDC inhibited RANKL-induced ERK activation without affecting JNK and p38 activation. The classical NF-*κ*B signaling pathway involves activation of the I*κ*B kinase (IKK) complex that stimulates I*κ*B*α* phosphorylation and degradation, allowing nuclear translocation of NF-*κ*B heterodimer containing the p50 and p65 subunits [20]. In addition to I*κ*B*α*, posttranslational p65 modifications such as phosphorylation, acetylation, and ubiquitination represent another mechanism regulating NF-*κ*B transcriptional activity [20]. It has been suggested that p65 phosphorylation at Ser536 in the transactivation domain promotes its transcriptional activity [21, 22]. In our study, WEDC suppressed RANKL-induced p65 phosphorylation at Ser536 and I*κ*B*α* phosphorylation and degradation. Therefore, our findings suggest that WEDC inhibits osteoclast differentiation, at least in part, by suppressing RANKL-induced activation of ERK and the classical NF-*κ*B signaling pathway, without interfering with the RANK-TRAF6 pathway.

In addition to the inhibitory effect on osteoclast differentiation, WEDC also decreased resorbed pit area by mature osteoclasts without affecting the number of osteoclasts, indicating that WEDC inhibits bone-resorbing activity of osteoclasts. Given the crucial role of actin ring structure in bone-resorbing activity of osteoclasts [19], our findings of WEDC-induced disruption of actin ring structure in osteoclasts suggest that the antiresorptive property of WEDC is due to disruption of actin ring structure. The classical NF-*κ*B signalling pathway is involved in bone-resorbing activity of osteoclasts as well as osteoclast differentiation. It has been shown that blocking NF-*κ*B pathway by overexpressing a dominant-negative form of IKK beta suppresses bone-resorbing activity of osteoclasts, whereas NF-*κ*B activation by expression of a constitutively active form of IKK beta upregulates it [23]. Neither inhibition nor activation of NF-*κ*B pathway by the IKK beta mutants affected osteoclast survival [23]. In addition, NBD peptide, a selective peptide inhibitor of the classical NF-*κ*B pathway, was shown to inhibit bone resorption by disrupting actin ring [24]. Therefore, these observations suggest that the inhibitory action of WEDC on the classical NF-*κ*B pathway may contribute to its antiresorptive function.

Consistent with the *in vitro* results, administration of WEDC protected bone loss induced by RANKL. Since injection of exogenous RANKL rapidly induces trabecular bone loss through stimulating osteoclast differentiation and function [17], the protective effect of WEDC on bone loss is mainly due to the suppression of osteoclast differentiation and function. However, we cannot exclude the possibility that WEDC might affect osteoblastic bone formation. Indeed, WEDC could stimulate matrix mineralization in calvarial osteoblast cultures (data not shown), although its effect on bone formation *in vivo* remains to be elucidated. Given the crucial role of excessive RANKL activity in pathological bone destruction, our findings strongly suggest that WEDC may be useful in preventing or treating various bone destructive diseases. 

Phloroglucinols, triterpenes, flavonoid glycosides, and phenolic compounds have been isolated from the rhizome of *D. crassirhizoma *[25–29]. Of them, phloroglucinol derivatives have been linked to several biological activities of the rhizome of *D. crassirhizoma*, including antioxidant, antibacterial, and antitumor activities [13, 25, 30]. We found the presence of a tetrameric phloroglucinol, dryocrassin ABBA, in WEDC with HPLC analysis (data not shown). Dryocrassin ABBA exhibited a marginal inhibitory effect on RANKL-induced osteoclast differentiation at concentrations without affecting cell viability (data not shown). However, the constituents mediating the protective effect of WEDC on bone loss still remain to be elucidated.

## 5. Conclusion

We have demonstrated that WEDC inhibits osteoclast differentiation and function by inhibiting RANK signaling pathways in osteoclast precursors and by disrupting actin ring in mature osteoclasts, respectively. Furthermore, WEDC suppressed RANKL-induced bone loss *in vivo*. These findings suggest that WEDC may be useful in preventing or treating bone diseases associated with excessive bone loss.

## References

[1] Boyle WJ, Simonet WS, Lacey DL (2003). Osteoclast differentiation and activation. *Nature*.

[2] Lacey DL, Timms E, Tan HL (1998). Osteoprotegerin ligand is a cytokine that regulates osteoclast differentiation and activation. *Cell*.

[3] Walsh MC, Kim GK, Maurizio PL, Molnar EE, Choi Y (2008). TRAF6 autoubiquitination-independent activation of the NF*κ*B and MAPK pathways in response to IL-1 and RANKL. *PLoS ONE*.

[4] Gohda J, Akiyama T, Koga T, Takayanagi H, Tanaka S, Inoue JI (2005). RANK-mediated amplification of TRAF6 signaling to NFATc1 induction during osteoclastogenesis. *EMBO Journal*.

[5] Huang H, Ryu J, Ha J (2006). Osteoclast differentiation requires TAK1 and MKK6 for NFATc1 induction and NF-*κ*B transactivation by RANKL. *Cell Death and Differentiation*.

[6] Hyung JK, Lee Y, Chang EJ (2007). Suppression of osteoclastogenesis by N,N-dimethyl-D-erythros-phingosine: a sphingosine kinase inhibition-independent action. *Molecular Pharmacology*.

[7] Ikeda F, Nishimura R, Matsubara T (2004). Critical roles of c-Jun signaling in regulation of NFAT family and RANKL-requlated osteoclast differentiation. *Journal of Clinical Investigation*.

[8] Yamashita T, Yao Z, Li F (2007). NF-*κ*B p50 and p52 regulate receptor activator of NF-*κ*B ligand (RANKL) and tumor necrosis factor-induced osteoclast precursor differentiation by activating c-Fos and NFATc1. *Journal of Biological Chemistry*.

[9] Iotsova V, Caamaño J, Loy J, Yang Y, Lewin A, Bravo R (1997). Osteopetrosis in mice lacking NF-*κ*B1 and NF-*κ*B2. *Nature Medicine*.

[10] Grigoriadis AE, Wang ZQ, Cecchini MG (1994). c-fos: a key regulator of osteoclast-macrophage lineage determination and bone remodeling. *Science*.

[11] Takayanagi H, Kim S, Koga T (2002). Induction and activation of the transcription factor NFATc1 (NFAT2) integrate RANKL signaling in terminal differentiation of osteoclasts. *Developmental Cell*.

[12] Bae KH (2008). *The Medicinal Plants of Korea*.

[13] Lee SM, Na MK, Na RB, Min BS, Lee HK (2003). Antioxidant activity of two phloroglucinol derivatives from *Dryopteris crassirhizoma*. *Biological and Pharmaceutical Bulletin*.

[14] Chang SH, Bae JH, Hong DP (2010). *Dryopteris crassirhizoma* has anti-cancer effects through both extrinsic and intrinsic apoptotic pathways and G0/G1 phase arrest in human prostate cancer cells. *Journal of Ethnopharmacology*.

[15] Yang Y, Lee GJ, Yoon DH (2013). ERK1- and TBK1-targeted anti-inflammatory activity of an ethanol extract of *Dryopteris crassirhizoma*. *Journal of Ethnopharmacology*.

[16] Kwon DY, Kang OH, Choi JG (2007). Antibacterial effect of *Dryopteris crassirhizoma* against methicillin-resistant *Staphylococcus aureus*. *Fitoterapia*.

[17] Tomimori Y, Mori K, Koide M (2009). Evaluation of pharmaceuticals with a novel 50-hour animal model of bone loss. *Journal of Bone and Mineral Research*.

[18] Lee JH, Kim HN, Yang D (2009). Trolox prevents osteoclastogenesis by suppressing RANKL expression and signaling. *Journal of Biological Chemistry*.

[19] Teitelbaum SL (2006). Osteoclasts; culprits inflammatory osteolysis. *Arthritis Research and Therapy*.

[20] Hayden MS, Ghosh S (2012). NF-kappaB, the first quarter-century: remarkable progress and outstanding questions. *Genes and Development*.

[21] Yang F, Tang E, Guan K, Wang CY (2003). IKK*β* plays an essential role in the phosphorylation of RelA/p65 on serine 536 induced by lipopolysaccharide. *Journal of Immunology*.

[22] Doyle SL, Jefferies CA, O’Neill LA (2005). Bruton’s tyrosine kinase is involved in p65-mediated transactivation and phosphorylation of p65 on serine 536 during NF-*κ*B activation by lipopolysaccharide. *Journal of Biological Chemistry*.

[23] Miyazaki T, Katagiri H, Kanegae Y (2000). Reciprocal role of ERK and NF-*κ*B pathways in survival and activation of osteoclasts. *Journal of Cell Biology*.

[24] Soysa NS, Alles N, Shimokawa H, Jimi E, Aoki K, Ohya K (2009). Inhibition of the classical NF-*κ*B pathway prevents osteoclast bone-resorbing activity. *Journal of Bone and Mineral Metabolism*.

[25] Lee HB, Kim JC, Lee SM (2009). Antibacterial activity of two phloroglucinols, flavaspidic acids AB and PB, from *Dryopteris crassirhizoma*. *Archives of Pharmacal Research*.

[26] Na M, Jang J, Min BS (2006). Fatty acid synthase inhibitory activity of acylphloroglucinols isolated from *Dryopteris crassirhizoma*. *Bioorganic and Medicinal Chemistry Letters*.

[27] Lee JS, Miyashiro H, Nakamura N, Hattori M (2008). Two new triterpenes from the rhizome of *Dryopteris crassirhizoma*, and inhibitory activities of its constituents on human immunodeficiency virus-1 protease. *Chemical and Pharmaceutical Bulletin*.

[28] Min BS, Tomiyama M, Ma CM, Nakamura N, Hattori M (2001). Kaempferol acetylrhamnosides from the rhizome of *Dryopteris crassirhizoma* and their inhibitory effects on three different activities of human immunodeficiency virus-1 reverse transcriptase. *Chemical and Pharmaceutical Bulletin*.

[29] Chang X, Li W, Koike K, Wu L, Nikaido T (2006). Phenolic constituents from the rhizomes of *Dryopteris crassirhizoma*. *Chemical and Pharmaceutical Bulletin*.

[30] Kapadia GJ, Tokuda H, Konoshima T, Takasaki M, Takayasu J, Nishino H (1996). Anti-tumor promoting activity of *Dryopteris* phlorophenone derivatives. *Cancer Letters*.

